# Post-surgical Comparison of Volumetric Analysis in Orbital Fractures with Conventional and Advanced Management

**DOI:** 10.1007/s12663-025-02754-3

**Published:** 2025-10-24

**Authors:** Luis Vicente González, María Paula Orjuela, Manuel Mejía, Gerardo Ardila-Duarte, Juan Pablo López

**Affiliations:** 1 Oral and Maxillofacial Surgeon, Hospital Universitario La Samaritana. , Bogotá, Colombia; 2 Oral and Maxillofacial Surgeon, Hospital Universitario Fundación Santa Fe de Bogotá, Bogotá DC, Colombia; 3 Escuela Colombiana de Carreras Industriales, Bogotá, Colombia; 4 Department of Oral Research, Institución Universitaria Colegios de Colombia, Bogotá, Colombia; 5 Universidad El Bosque, Unidad de Investigación en Epidemiología Clínica Oral UNIECLO, Calle 129#7d-47, Bogotá DC, Colombia

**Keywords:** Orbital fractures, Virtual planning, 3D printing, Endoscopy, Virtual navigation

## Abstract

**Aim:**

This study aims to compare the volumetric changes of the orbit after using conventional technique or virtual navigation endoscopically integrated and virtual planning in orbital trauma.

**Methods:**

This is an analytical prospective cohort study. The sample was subjects that underwent primary reconstruction for unilateral orbital deformities secondary to trauma. The population was divided into three groups: A control group in order to establish in our patients a reference of the standard difference between both orbits (Group 1). The conventional technique (Group 2) and virtual navigation endoscopically integrated combined with virtual planning (Group 3). Both groups were compared separately with the control group.

**Results:**

In total, 18 patients were divided into three groups. In Group 2, the differential between the orbits was 6.31. In Group 3, the differential between both orbits was 1.14 finding statistically significant differences between Group 1 and Group 2 (*p* = 0.02), and between Group 2 and Group 3 (*p* = 0.03). Additionally, between Group 1 and Group 3 no statistically significant differences were found (*p* = 0.10).

**Conclusion:**

Combining these two techniques into the same instrument for orbital reconstruction can be a great alternative and can be useful to decrease the risk of complications associated with this procedure and improve the results and sequelae.

## Introduction

The relationship between the increase in orbital volume and the herniation of the orbit’s contents due to fracture of the different walls and malar fractures is established [1, 2]. However, orbital reconstruction is challenging due to its complex anatomy, limited visualization, and asymmetry compared to the contralateral orbit [3].

To date, multiple techniques have been developed and combined to improve results. Endoscopic surgery offers greater visualization, which helps minimize damage to relevant anatomical structures and facilitates greater precision [4]. Virtual navigation gives instant understanding of the position of the instruments with respect to the patient’s anatomy. Its main advantage is the ability to recognize the exact orbital dimensions and limits in all planes of space. This allows the surgeon to achieve an ideal position for the graft or material used in the reconstruction [5].

In this study, various techniques mentioned were integrated into a single instrument, facilitating their use and improving clinical results. Additionally, the orbital volume is considered, as it is crucial to restore it accurately to avoid complications. Therefore, the study is justified, as the use of these techniques separately, but never unified, has been previously published, comparing them with the conventional method in relation to orbital volume.

This study aims to compare the volumetric changes of the orbit after using either the conventional technique or virtual navigation endoscopically integrated, combined with virtual planning, in the treatment of orbital trauma.

## Materials and Methods

This is a descriptive observational study with a prospective cohort study in subjects that underwent primary reconstruction for orbital fractures who attended the Hospital Universitario de la Samaritana, Bogotá DC, Colombia, with ethical approval 04-2020. Patients were operated on by the same surgeon between 2018 and 2019. Informed consent was obtained from the patient. Subsequently, the patients were randomly assigned to the groups using the block randomization technique. The website random.org was used.

A control group (Group 1) was established, randomly selected from CT scans taken for other diagnostic purposes in patients without orbital fractures. Subjects were randomly assigned to two groups. One of the groups included subjects managed with the conventional technique (Group 2), and the other (Group 3) included subjects treated with the assistance of endoscopic surgery and integrated virtual navigation and planning. The diagnosis of an orbital fracture was made based on clinical evaluation and CT findings obtained. All procedures were ambulatory, with the prescription of antibiotic therapy and NSAID pain relievers.

The data were extracted from the study population’s records. The independent variables were orbital surgery performed with either a conventional technique or with endoscopic support and virtual surgical planning. The dependent variables, extracted from the data, were classified as orbital volume.

The inclusion criteria were subjects over 18 years of age with a pure unilateral orbital floor fracture, with a postoperative computed tomography (CT). Exclusion criteria included subjects with inadequate postoperative CT scans and multiple walls involved. The participants were asked to complete a questionnaire that included their age (in years) and gender (male or female).

The decision to treat was made if the function or aesthetic was altered (extraocular muscle entrapment, eye movement, and diplopia). The function was evaluated clinically employing tests of visual field, ocular movement, campimetry, and presurgical and postsurgical Hertel or Naugle exophthalmometry. Two independent, blinded maxillofacial surgeons evaluated the aesthetics.

### Computer Tomographic Analysis

The orbital volume was evaluated using MIMICS 17.0 software (Materialise NV, Leuven, Belgium) by a biomedical engineer who was unaware of the technique used and the study’s objective. The engineer employed control points obtained through manual inspection at the limits of the eyeball in the sagittal plane. This process was carried out every three cuts (1.8 mm) for an average of 10 planes, each with approximately eight control points. This was carried out for both the right and left eyeballs; this protocol is executed in the diagnostic imaging module of the 3D SLICER software.

To create the volume from serial planes with the control limit area, it was necessary to connect these control points, identify a plane perpendicular to it (coronal), and then create the control limit area on that plane. Once done, it generates the joining of the sagittal planes, following the contour of the coronal plane area, and a guide plane sweep operation**.**

Once the volumes were generated, their edges were smoothed (union of the flat plane with the sagittal plane) using the WRAP tool in the software. This smoothing can cause certain areas to leave the initial control area; to guarantee that the volume really contains the information of the eyeball, this smoothed volume is taken and a BOOLEAN subtraction operation is performed, between the control volumes and the segmented skull; finally, they are manually verified that the volumes are inside the eyeballs; with respect to the anterior zone of these volumes, their extension, the ocular curvature, controlled it, visually noticeable in the sagittal plane of the CAT. This is important to prevent bias from the information.

### Surgical Technique

Surgical access for Group 2 was made through a transconjunctival approach and a lateral canthotomy, depending on the size of reconstruction. Second, an intraoral incision was made in the maxillary buccal sulcus, from approximately 2 cm on the side of the fracture, and then, an osteotomy was performed. On occasion, a fracture of this maxillary bone may occur, and a window antrostomy should be designed using a Kerrison rongeur for bone removal. Under the endoscope view, the maxillary sinus was scrutinized.

**Fig. 1:**
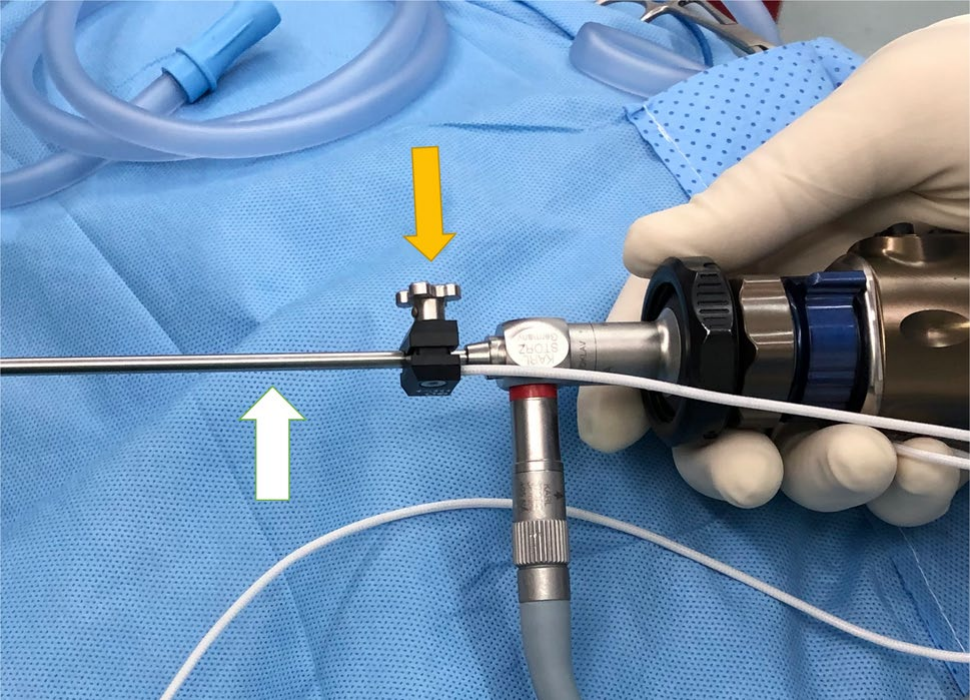
Endoscopic integration with virtual navigation in the same instrument

**Fig. 2:**
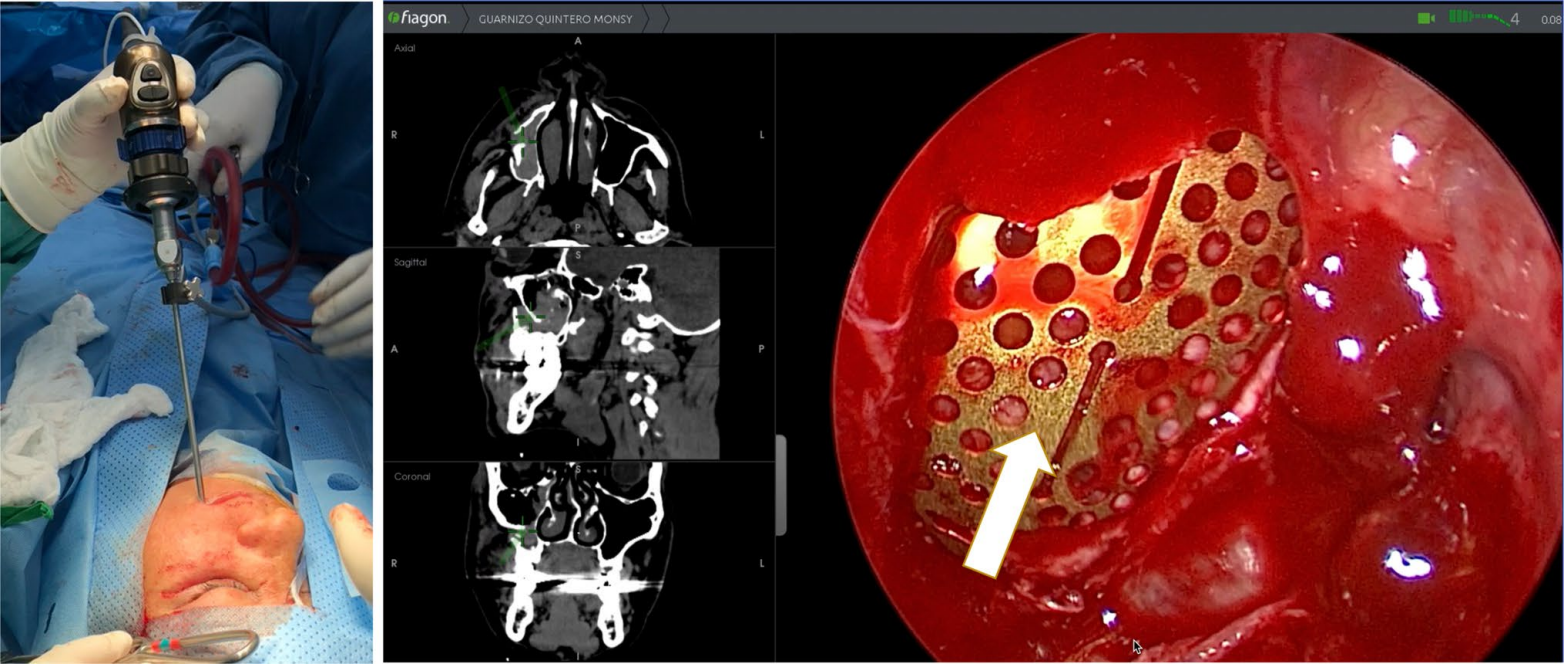
Virtual navigation with endoscope integration through the maxillary sinus, checking the final position of the orbital mesh

The endoscope is integrated with navigation within the same instrument to facilitate its manipulation (Fig. 1). The next step was to remove the mucosa of the sinus to expose the area and determine the fracture’s size, degree of fat herniation, bony comminution, and muscle entrapment. Additionally, the reconstruction material is selected based on the size of the defect. It is inserted through the orbital approach under endoscopic and virtual navigation (Fiagon ENT Navigation System, Berlin, Germany)**—**Fig. 2. Therefore, the posterior boundary of the orbital floor is directly controlled during the entire positioning of the mesh to get a safe and accurate final position. Otherwise, the surgical access for Group 3 was made through a transconjunctival approach and probably a lateral canthotomy. The orbital floor defect was reconstructed without any endoscopic support; moreover, it was only at one point that this approach allowed for visual control.

### Other Variables

The operative time was measured from the beginning of the anesthetic infiltration to the end of the suture and was recorded in minutes, yielding an average for the procedures. Additionally, patient satisfaction was measured through a multiple-choice survey. It was explained to patients that satisfaction should encompass the overall outcome of the procedure, without considering other factors, such as hospital stay.

### Analysis Plan

All the data were analyzed using the Real Statistics V8.1q. Initially, an exploratory analysis was performed to describe the sample, including measures of central tendency. The Shapiro–Wilk test was used to determine if the demographic data were normally distributed. The difference between both orbits in each of the groups was determined, and subsequently, an ANOVA I test was performed to compare the differentials between the groups studied. Finally, the post hoc Tukey ANOVA I test was applied to verify which of the groups presented the least difference compared to the control group.

## Results

The study population consisted of 18 patients, divided into three groups, each with 6 subjects. The largest population was male (n = 16), and the mean age of the patients was 36.5 years. Before surgery, the most frequent characteristic in this study was diplopia (n: 7), followed by enophthalmos (n: 4). The most frequent cause of fractures was physical aggression (n: 9), followed by traffic accidents (n: 3). The left orbit was found to be compromised in 8 subjects and the right orbit in 4. It was determined that the groups are comparable to each other, having a normal distribution, as indicated by the Shapiro–Wilk test (*p* > 0.05)**—**Table 1.

**Table 1:** Summary of demographic information

Group	Subjects	Mean age	Gender		Cause of injury	Side of injury		Diplopia/ Enophtalmos	Operative time (Mean)	SW *p*-value
			Male	Female		Left	Right			
Group 1	6	35.6	6	0	N/A	N/A	N/A	N/A	N/A	0,90 0,91
Group 2	6	38.5	5	1	Physical aggression: 4 Traffic accident: 2	3	3	Diplopia: 2 Enophtalmos: 4	53 min	0,25 0,63
Group 3	6	35.4	5	1	Physical aggression: 5 Traffic accident: 1	5	1	Diplopia: 2 Enophtalmos: 3	97 min	0,94 0,95
Total	18	36.5	16	2	Physical aggression: 9 Traffic accident: 3	8	4	Diplopia: 4 Enophtalmos: 7	N/A	

N/A, Not applicable; min, minutes

The mean difference between both orbits in the control group (Group 1) was 0.8. The group treated with the conventional technique (Group 2) showed an average volume in the non-fractured orbit of 29.67 and in the fractured orbit of 35.98, resulting in a mean difference of 6.31 between the two orbits. Regarding the group treated with the advanced technique (Group 3), the mean volume for the non-fractured orbit was 26.25, and for the fractured one, it was 27.39, with a mean difference of 1.14 between the two orbits. In Group 2, when patient satisfaction with the procedure was evaluated, 3/6 patients were not satisfied, which may be due to enophthalmos. On the other hand, in Group 3, the patients predominantly reported being very satisfied (4/6). Two subjects in Group 3 were poorly satisfied or not satisfied due to sequelae, such as ectropion, despite not presenting any visible sequelae and despite the orbital volume being adequately restored—Table 2.

**Table 2:** Summary of the orbital volume in different groups

Group 1	Right orbit	Left orbit	Difference between orbits	Patient satisfaction	Mean difference
Patient 1	29.50	28.89	0.61	N/A	0.8
Patient 2	33.69	34.32	0.62	N/A	
Patient 3	32.29	32.25	0.04	N/A	
Patient 4	30.34	29.77	0.57	N/A	
Patient 5	35.08	36.86	1.78	N/A	
Patient 6	29.67	28.48	1.19	N/A	

Group 2	Fractured Orbit	Non-fractured orbit	Difference between orbits	Patient satisfaction	Fractured orbit
Patient 1	29.91	27.37	2.54	Satisfied	6.31
Patient 2	31.93	29.03	2.90	Poorly satisfied	
Patient 3	46.88	30.28	16.60	Not satisfied	
Patient 4	32.60	26.24	6.36	Not satisfied	
Patient 5	40.32	32.73	7.59	Not satisfied	
Patient 6	34.25	32.39	1.86	Very satisfied	

Group 3	Fractured Orbit	Non-fractured orbit	Difference between orbits	Patient satisfaction	Fractured orbit
Patient 1	24.56	23.69	0.87	Not satisfied	1.14
Patient 2	23.78	22.99	0.79	Very satisfied	
Patient 3	26.34	25.9	0.44	Very satisfied	
Patient 4	28.77	27.99	0.78	Very satisfied	
Patient 5	32.68	29.67	3.01	Poorly satisfied	
Patient 6	28.18	27.23	0.95	Very satisfied	

**Fig. 3:**
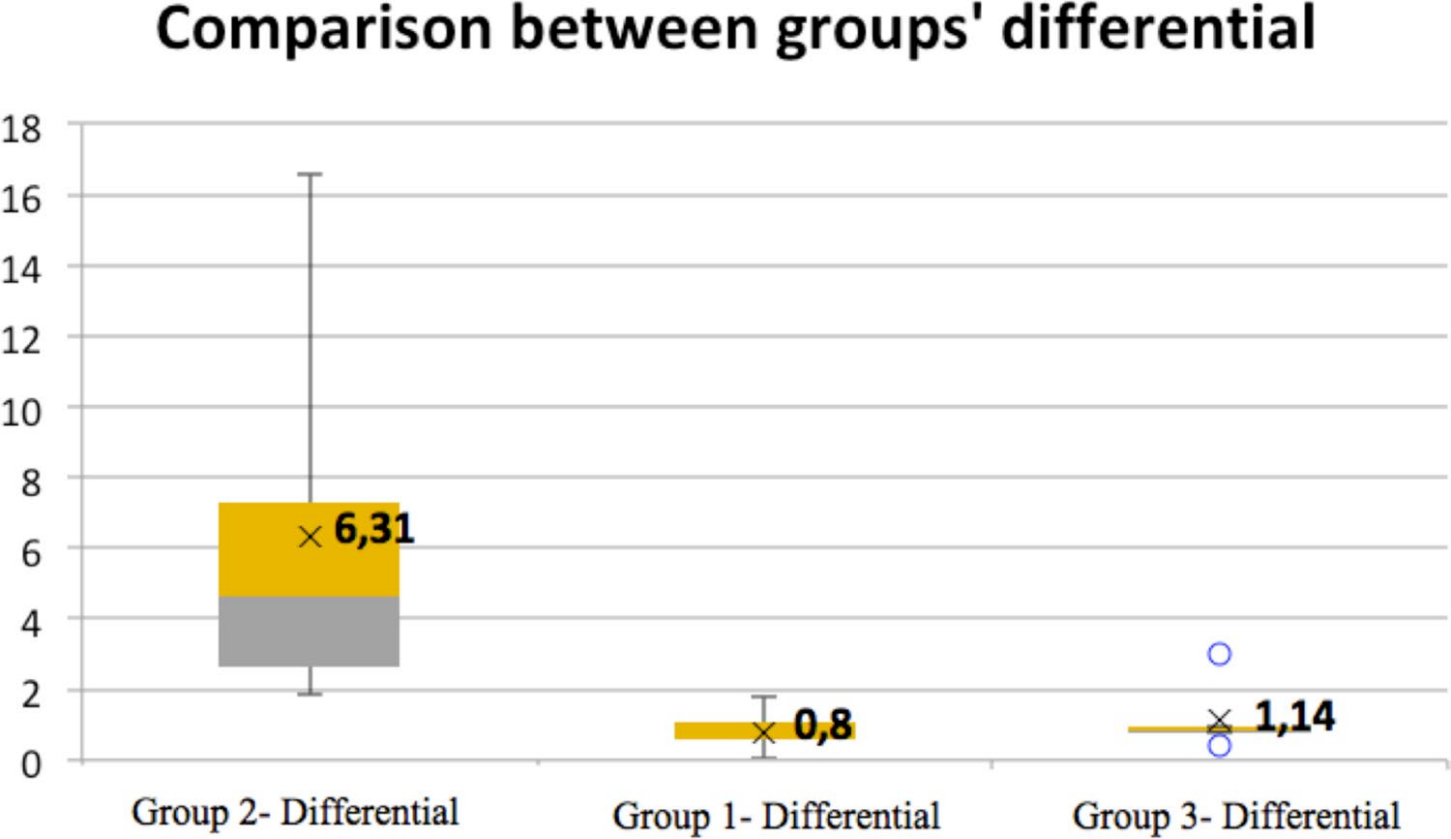
Comparative analysis between the groups comparing orbital volume

Regarding the distribution of the volume differentials in each of the groups, it was observed that Group 3 had the most uniform distribution, with significantly smaller differences between the two orbits compared to Group 2 and the lowest mean differential. However, this group presented certain outliers above and below the normal distribution (0.44 and 3.01). Group 2 presented an abnormal and asymmetric distribution, with a mean differential greater than that of Groups 1 and 3**—**Fig. 3.

Comparisons were made between the means of the differentials of the orbital volumes of each of the groups with each other. Statistically significant differences were found in the comparison between Group 1 and Group 2 (*p* = 0.026) and in the comparison between Group 2 and Group 3 (*p* = 0.037). In contrast, the comparison between Group 1 and Group 3 did not reveal statistically significant differences (*p* = 0.98)—Table 3.

**Table 3:** Comparison of outcomes of the groups

Comparison between groups		ANOVA I *p*-value	Mean difference	Std err	*p*-value
Group 1	Group 2	0.0173	5.50	1.33	0.026 *
Group 2	Group 3		5.16	1.33	0.037 *
Group 1	Group 3		0.33	1.33	0.982

Group 1: Control

Group 2: Conventional technique

Group 3: Advanced technique

*Statistically significant

## Discussion

Several clinical criteria help the clinician to assess the correct position of the eyeball after orbital trauma. However, these criteria provide a purely subjective judgment about this condition. Therefore, it is essential to consider more objective variables, such as orbital volume, to determine the precise position of the eyeball and achieve better results in patients who have undergone orbital reconstruction. The normal orbital volume ranges from 23 to 25 ml, depending on age [6]. Regarding gender, the volume for men is 24.9 ± 3.03 cc, and for women, it is 23.9 ± 3.08 cc [7]. The differences that exist between the orbits can be as small as 0.8 mL [8]. Regarding the variations in this volume in patients who have suffered trauma to the orbit, it has been determined that in blow-out fractures, there may be an increase of up to 121.46% associated with the location of the fracture.

Orbital volume has been demonstrated as a reference for determining postoperative changes in orbital reconstruction, compared to the Hertel scale, which is also a quantitative measure [9]. Choi et al. determined that the presence of enophthalmos increases proportionally with the increase in orbital volume, with a statistically significant relationship between this orbital volume and the degree of enophthalmos [10]. A systematic review found a direct relationship between orbital volume and enophthalmos, concluding that for every 1 cm^3^ increase in volume, there can be an increase of 0.8 mm of enophthalmos [11]. Additionally, a combination of factors, including fracture area, muscle involvement, orbital volume, and fracture depth, may better predict the likelihood of functional sequelae [12]. Additionally, the volume of herniated orbital content has been correlated with the fractured area. It is also an important, although not the only, factor to consider when making decisions in orbital trauma. However, the portion that was directly related to the prediction of enophthalmos was the herniated tissue when it occurs posterior to the vertical equator of the eyeball [13]. Concerning diplopia, Safi et al. demonstrate the positive and significant association between the volume of tissue herniated with respect to the pre- and postoperative diplopia.

Additionally, they highlight the relationship between this volume and the persistence of diplopia (*p* = 0.04) [14]. These findings support that the presence of herniated tissue can influence the persistence of diplopia. This is attributed to the fact that these fractures have a greater likelihood of causing muscle entrapment, resulting in diplopia [15]. The presence of enophthalmos and diplopia can significantly impact a patient’s quality of life. Therefore, alterations in orbital volume and the presence of these factors should be recommended to improve the diagnosis and treatment of orbital fractures.

The technological tools have contributed to the control of the variables under discussion. Surgical planning, intraoperative navigation, and endoscopic devices, as demonstrated in our study, can be beneficial. A study incorporating intraoperative navigation and stereolithographic models to reconstruct fractured orbits yielded satisfactory results, with a difference between orbits of less than 1 cm^3^ and complete resolution of diplopia [16]. Alternatively, navigation and endoscopic support are also of great assistance in achieving a better result. Navigation helps reconstruct images where anatomical landmarks have been broken, and endoscopic support helps to visualize defects more accurately. Both technologies have been shown to improve outcomes [17]. In our study, the combined use of technologies such as surgical navigation and endoscopic surgery enabled us to achieve results with volumes differing by less than 1 cm^3^ in both orbits. These results were very similar to those of the control group, which showed differences of 0.8 cm^3^ between the two orbits. In turn, when comparing this technique with the conventional method, better results were observed with the use of the advanced technique. One of the difficulties with these two technologies has been the separate handling of the two instruments. For this, an article proposed the use of navigation endoscopically integrated, which helped to combine the two technologies into a single instrument, thereby improving the procedure’s results [18].

## Conclusion

Combining these two techniques into a single instrument for orbital reconstruction can be a valuable alternative, reducing the risk of complications associated with this procedure while also enhancing the results and outcomes. Morbidity in Group 3 may be higher if the need to perform an additional approach through the maxillary sinus is considered; however, reducing the possibility of reinterventions can make the technique less morbid in the long term.
